# Action of Alcohol as an Aliment in Disease

**Published:** 1862-08

**Authors:** 


### ACTION OF ALCOHOL AS AN ALIMENT IN DISEASE.

Dr. F. E. Anstie, Assistant Physician to the Westminister Hospital relates, (*London Medical Review*, April, 1862,) four cases, one of pericarditis, two of pneumonia, and one of pleurisy, illustrative of the action of alcohol as an aliment in disease. These cases, he states, "are by no means the only ones which I could quote from my own practice, which might serve to throw light on the question whether alcohol does or does not act as a foyd. I have selected them, because they are the only cases of which I possess notes in which a crucial experiment was performed, viz: the administration of no food (for none other could be retained on the stomach) but alcohol, in the shape of wine or spirit, mixed with a little water. I think my readers must allow that there is no imperfection in the conditions of the experiments which is at all important; and if this be so, the evidence which they afford is very important. It may be briefly summed up thus:

"1. Alcohol is capable of sustaining life, in the absence of all other foods, for many days.

"2. During acute diseases, alcohol is sufficient, without the use of any food, to prevent emaciation of the body, and also the extreme lowering of muscular strength, which would render the period of convalesence tedious.

"3. Given in acute disease no amount of alcohol which the exigencies of the case require will cause inebriation; on the contrary, delirium, unless it depend on inflammation of the brain, may be always checked by the administration of alcohol.

"That the demand of the system for alcohol in acute disease is in inverse ratio to the power of assimilating other foods.

"To these conclusions I may add some others, to which I am led, not by the above cases only, but by a very large number of others also.

"1. It is not true that alcohol may only be given with advantage, in acute disease, when the skin is already moist and perspiring. On the contrary, nothing has been more common in my experience than to see patients whose skin had previously been dry and burning hot, break out into a refreshing sweat after some hours of steady perseverance in the administration of alcohol in two drachm doses every half hour.

"2. It is not true that the treatment of acute disease by large doses of alcohol involves any danger whatever of laying the foundatian of habits of drunkenness. If the alcohol has been judiciously administered, in sufficient quantities, and yet not too large, the moment that the appetite for ordinary food returns the desire for the temporary substitute will cease. I fully agree with the important remarks recently made on this subject by Dr. Druitt. It may be laid down as a rule that, both in disease and in health, the taking of so large a quantity of alcohol as, under the circumstances, is sufficient to produce intoxication, tends to produce a craving for the stimulus; and it is equally certain that the timid and inadequate use of stimulants in acute diseases tends to produce a similar *bulimia*. But the taking of even a bottle or two bottles of brandy a day, when the case requires it, does not engender the smallest taste for alcohol after the return of health. And the daily use, during health, of a quantity of alcohol which is insufficient to produce any, even the earliest, symptoms of inebriation, does not occasion the smallest craving for an increase of the dose."—*Am. Med. Jour.*

